# Hydatid Cyst of the Rib: A New Case and Review of the Literature

**DOI:** 10.1155/2009/817205

**Published:** 2010-02-21

**Authors:** A. Chafik, A. Benjelloun, A. El Khadir, R. El Barni, A. Achour, M. A. Ait Benasser

**Affiliations:** ^1^General Surgery Unit, Hôpital Militaire Avicenne, Marrakech, Morocco; ^2^Pulmonology Unit, Hôpital Militaire Avicenne, Marrakech, Morocco

## Abstract

The hydatid cyst is not rare in our country, but bone lesions are less common. The disease often takes the appearance of abscess or malignant lesion. We report a case of a 35-year-old man with a hydatid cyst of the rib complicated with cutaneous fistula. The surgery allowed both diagnosis and treatment. Albendazole was then administered to prevent relapse.

## 1. Introduction

The hydatid cyst is a parasitosis caused by Echinococcus granulosus, a cestode which remains endemic in some parts of the world.

This study is relevant to Morocco because of the exposure of its rural population to infection due to their proximity to the carnivores, sheep, and bovins. Liver (60%) and lungs (20% to 30%) are the most affected by the disease [1]. Osseous hydatidosis is uncommon (0.9% to 4%) especially the ribs [2]. Bone lesions are always primary; secondary lesions are due to recurrence.

In this case, we present a patient with costal echinococcosis that looks like—as revealed by a cutaneous fistula—an infectious lesion. The course of the disease is usually slow and laboratory tests are often negative. Diagnosis is generally made through the combined assessment of clinical, radiologic, and laboratory data.

## 2. Case Report

A 35-year-old man was admitted to our unit for a skin fistula on the left chest wall. He was a moderate smoker who had no history of substance abuse. Five years ago, after a parachute jump, he had suffered from chest pain with swelling of the left chest wall and a broken rib. The swelling was drained in emergency room, but left a cutaneous fistula without accurate diagnosis.

On admission, physical examination showed a palpable mass at the left 6th rib's level, with no other abnormality. Blood cell count, hydatid serology, and inflammation markers were normal.

**Figure 1:**
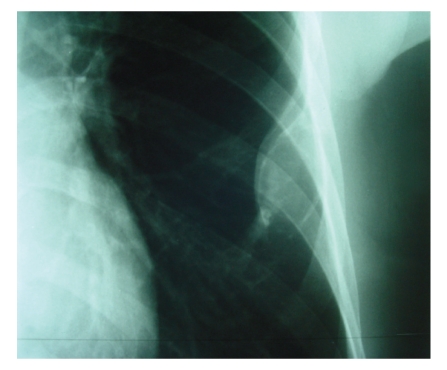
Chest radiography : Ovoïd opacity of the lateral arch of the 6th left rib.

**Figure 2:**
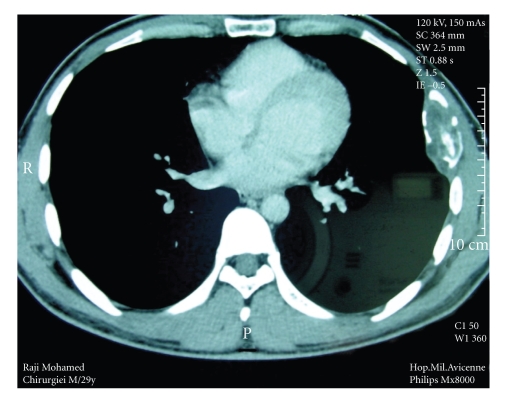
Chest CT scan: tumor of the 6th left rib without invasion of adjacent tissues.

Chest X-rays showed an opacity on the 6th left rib (Figure 1). A computed tomography confirmed the opacity on the 6th left rib with a cortical break without any invasion of the adjacent structures (Figure 2). The lung parenchyma and mediastinum were free.

The patient underwent a thoracotomy. An incision was performed along the lateral arch of the 6th rib, where we noticed a hydatid vesicle and bone sequesters. We resected the lateral arch of the 6th rib on a 10 cm length. There were no postsurgical complications. Pathological analysis revealed a hydatid cyst of the rib. A medical treatment was prescribed to the patient (Albendazole 400 mg/day 6 months). 18 months after this surgery the patient is still in good condition and has no cyst relapse.

## 3. Comment

Costal hydatid disease is very rare, even in the countries where the disease is endemic. In rib lesions, hydatid cyst destroys the bone matrix and usually infiltrates adjacent tissues [3], which, fortunately, has not happened in our specific case. The disease evolution is generally slow but it is important to note that complications can occur. The diagnosis is usually suspected based on the conditions of life and the radiological aspect. Serological tests might help in the diagnosis, but one has to keep in mind that the best sensitivity barely reaches 82.7% and the best specificity 94.7% [4]. Some authors recommend also CT scan and even ultrasound [5].

Ruptured and infected hydatid cysts are often confused with tumors and/or abscesses [6].

Early diagnosis is important to prevent complications. When an intrathoracic extrapulmonary hydatid cyst lies in the neighborhood of bone structures, it can cause bone destruction. Rupture of a pulmonary hydatid cyst into the pleural space, either spontaneously or during surgery, is the most common cause of pleural hydatidosis or chest wall hydatidosis.

In this case, the hydatid cyst is primary; it involved neither the lung nor the liver. The possible mechanism of primary hydatid disease of the chest wall may be as follows: the embryo passes through the duodenal wall into either the portal vein or the periduodenal and perigastric lymphatics. Periduodenal and perigastric lymphatic channels connect with the thoracomediastinal lymphatic and the thoracic duct [7].

The gold standard is to perform surgery in excising the entire rib and to use pre- and postoperative medical treatment [8]. It has been suggested that better results would be achieved by combining surgery and albendazole (10 mg/kg) for presurgery and postoperative prophylaxis, and that large doses over a long period of time would be a good clinical approach and may reduce the incidence of relapse [9]. Our preference goes to surgery first followed by 6 months of postoperative prophylaxy by albendazole 400 mg/day, with monthly hepatic balance monitoring, given the risk of hepatotoxicity.

Hydatid cyst, especially of the rib, is a very rare disease. However the reported case demonstrates the importance of this differential diagnosis.

In conclusion, what makes this case special is the remarkably late onset of the disease. Given the patient's history, one might have been led to a wrong diagnosis, especially taking into account that radiology was inconclusive.

## References

[1] Tomos P, Kakaris S, Lachanas E, Karakatsani A (2005). Secondary echinococcosis of the rib and soft tissues. *Respiration*.

[2] Gezer S, Altinok T, Agaçkira Y, Tastepe I (2007). Hydatid disease of the first rib causing thoracic outlet syndrome. *Medical Principles and Practice*.

[3] Karaog lanoglu N, Gorguner M, Eroglu A (2001). Hydatid disease of rib. *Annals of Thoracic Surgery*.

[4] Stoss S, Kalbermatten DF, Robertson A (2008). Large cystic tumour at the chest wall mimicking an echinococcosis: a case report. *Journal of Plastic, Reconstructive and Aesthetic Surgery*.

[5] Ousehal A, Adil A, El Azhari A, Kadiri R (1995). Spinal cord compression disclosing rib hydatidosis. *Journal de Radiologie*.

[6] Kilic D, Tercan F, Sahin E, Bilen A, Hatipoglu A (2006). Unusual radiologic manifestations of the echinococcus infection in the thorax. *Journal of Thoracic Imaging*.

[7] Findikcioglu A, Kilic D, Hatipoglu A (2007). Primary hydatid disease of the chest wall. *Annals of Thoracic and Cardiovascular Surgery*.

[8] Stamatis G, Greschuchna D (1989). Echinococcus cysticus costalis: report of 2 cases and review of the literature. *Pneumologie*.

[9] Di Gesu G, Massaro M, Picone A, La Bianca A, Fiasconaro G (1987). Bone echinococcosis. *Minerva Medica*.

